# Expiratory braking defines the breathing patterns of asphyxiated neonates during therapeutic hypothermia

**DOI:** 10.3389/fped.2024.1383689

**Published:** 2024-05-20

**Authors:** Paola Papoff, Elena Caresta, Benedetto D’Agostino, Fabio Midulla, Laura Petrarca, Luigi Giannini, Francesco Pisani, Francesco Montecchia

**Affiliations:** ^1^ Pediatric Intensive Care Unit, Department of Pediatrics, Sapienza University of Rome, Rome, Italy; ^2^ Pediatric Emergency Care, Department of Pediatrics, Sapienza University of Rome, Rome, Italy; ^3^ Pediatric Neurology, Department of Pediatrics, Sapienza University of Rome, Rome, Italy; ^4^ Child Neurology and Psychiatry Unit, Department of Human Neurosciences, Sapienza University of Rome, Rome, Italy; ^5^ Medical Engineering Laboratory, Department of Civil Engineering and Computer Science, University of Rome “Tor Vergata”, Rome, Italy

**Keywords:** breathing pattern, esophageal pressure, expiratory braking, hypoxicischemic encephalopathy, neonate, respiratory effort, respiratory flow, respiratory mechanics

## Abstract

**Introduction:**

Although neonatal breathing patterns vary after perinatal asphyxia, whether they change during therapeutic hypothermia (TH) remains unclear. We characterized breathing patterns in infants during TH for hypoxic-ischemic encephalopathy (HIE) and normothermia after rewarming.

**Methods:**

In seventeen spontaneously breathing infants receiving TH for HIE and in three who did not receive TH, we analyzed respiratory flow and esophageal pressure tracings for respiratory timing variables, pulmonary mechanics and respiratory effort. Breaths were classified as braked (inspiratory:expiratory ratio ≥1.5) and unbraked (<1.5).

**Results:**

According to the expiratory flow shape braked breaths were chategorized into early peak expiratory flow, late peak expiratory flow, slow flow, and post-inspiratory hold flow (PiHF). The most braked breaths had lower rates, larger tidal volume but lower minute ventilation, inspiratory airway resistance and respiratory effort, except for the PiHF, which had higher resistance and respiratory effort. The braked pattern predominated during TH, but not during normothermia or in the uncooled infants.

**Conclusions:**

We speculate that during TH for HIE low respiratory rates favor neonatal braked breathing to preserve lung volume. Given the generally low respiratory effort, it seems reasonable to leave spontaneous breathing unassisted. However, if the PiHF pattern predominates, ventilatory support may be required.

## 1 Introduction

Moderate therapeutic hypothermia (TH) and optimized intensive care are recommended treatments for neonates with moderate to severe hypoxic-ischemic encephalopathy (HIE) to reduce mortality and long-term neurological disability (1).

Respiratory management is critical in neonates with HIE because both asphyxia and hypothermia can impair respiratory function (2). HIE may be associated with an abnormal respiratory drive, resulting in periodic breathing or apnea (3). Hypoxia often interrupts expiration leading to expiratory braking (4). Additionally, HIE can cause respiratory failure due to meconium aspiration, surfactant inactivation, and increased pulmonary vascular resistance (2). On the other hand, TH markedly depresses respiratory function, directly by altering the neuronal and chemorecptor activity (5), and indirectly, by reducing oxygen consumption and carbon dioxide production (6). TH may also adversely affect respiration by increasing pulmonary vascular resistance (7), decreasing respiratory muscle function (8) and oxygen release (9). As TH is associated with pain and stress, continuous low-dose opioids are often used to ensure neuroprotection. Opioids can cause apnea, bradypnea and have an effect on tidal volume, either increasing or decreasing it depending on the dose (10). Anticonvulsants are also used in some babies to prevent brain damage or to treat seizures. They can also affect breathing, for example benzodiazepines increase respiratory rate and decrease tidal volume (11), while fenobarbital may increase tidal volume (12). To prevent respiratory complications, it is standard practice for many resuscitated infants to receive mechanical ventilation throughout the TH despite adequate respiratory drive. Many centers that have expanded their cooling protocol to include mild HIE report a low rate of mechanical ventilation because of the perceived lower risk of respiratory complications in less severe HIE (13). In our practice, neonates with mild or moderate HIE who are successfully resuscitated usually avoid mechanical ventilation, which is initiated according to the needs of each patient once effective ventilation is established.

The respiratory pattern in healthy infants is age-dependent (14–18). Expiratory airflow patterns in neonates in the first few hours after birth indicate significant braking (14–17). Expiratory braking helps to maintain the end-expiratory lung volume (EELV) above the respiratory system relaxation volume (16, 17). This maximizes the time for pulmonary gas exchange and by preventing lung collapse averts atelectasis (17). Two braking mechanisms that contribute to maintaining an elevated EELV are post-inspiratory diaphragmatic activity, which reduces the rate of lung deflation by counteracting the passive elastic recoil of the respiratory system toward the relaxation volume, and upper airway narrowing at the level of the vocal cords, which increases the expiratory resistance to airflow and allows the airway pressure to exceed the atmospheric pressure (17, 19). Overall, neonatal breathing patterns show slow deep breaths soon after birth, more rapid and shallow breathing up to tachypnea within 90 min and, after a few days, breathing stabilizes at levels between those observed in the first few minutes and tachypnea (20).

Except for data on respiratory flow patterns in the first few days after uncomplicated birth (14, 15), there is no evidence from the neonatal period that can be used to predict the flow pattern during TH. Understanding the respiratory pattern of neonates cooled for HIE may provide useful information about the safety of spontaneous breathing or the need for respiratory support during TH.

The aim of this study was to investigate the breathing patterns of neonates with HIE during TH and subsequent normothermia and to categorize these patterns according to their flow behavior. We also examined potential differences in respiratory variables. To provide information on respiratory mechanics and breathing effort we also recorded esophageal pressure (Pes). For comparison, we studied infants with HIE who did not receive TH.

## 2 Materials and methods

### 2.1 Study population

We enrolled spontaneously breathing neonates with HIE who met the criteria recommended for TH by the Italian Society of Neonatology, which included gestational age ≥36 weeks, postnatal age <6 h, condition (A) 10-min Apgar score of ≤5; or continued need for resuscitation at 10 min after birth; or fetal acidosis: pH of <7.00 or base excess (BE) of ≤−12 mmol/L (within 60 min of birth), and condition (B) moderate or severe HIE as assessed by Sarnat score within 30 and 60 min of birth (Sarnat 2, moderate, Sarnat 3, severe) (21).

We also enrolled neonates with mild HIE, a condition preceded by a sentinel event before labor or delivery and often corroborated by a BE of minus 12 mmol/L (within an hour of birth). Mild HIE is characterized by a Sarnat score of 1 (hypervigilance, normal tone and activity, exaggerated Moro reflex, and normal autonomic function) on initial clinical examination. The decision to treat neonates with mild HIE with TH was left to the Pediatric Intensive Care Unit (PICU) physician clinical judgment.

Neonates with HIE who did not meet all criteria needed for TH were enrolled as controls. If incomplete conditions A or B were confirmed upon arrival to the PICU and the amplitude integrated electroencephalogram (aEEG) features were normal, TH was abandoned. Neonates were excluded at any time if they had respiratory distress, signs of infection, seizures, or required ventilatory support. Neonates were also excluded if they had genetic malformations or conditions other than perinatal asphyxia. The study was approved by the Ethics Committee of the Policlinico Umberto I, Sapienza University of Rome (protocol number 4089.2021). All neonates enrolled in the study were outborn. Consent for TH was obtained from the referral center, while consent for the study was obtained from a parent upon the neonate's admission to PICU.

Demographic, clinical, and instrumental data were collected from the medical records. All neonates studied underwent aEEG (OLYMPIC CFM 6,000, München, Germany) during TH, MRI scan at approximately one week of life and a standard EEG on day 10 of life. Neurological evaluations were performed daily using the Sarnat score. In accordance with the standard of care, neonates undergoing TH were routinely started on a continuous infusion of fentanyl at 1 mcg/kg/h modulated according to the Neonatal Infant Pain Scale (NIPS, target 0–2) (22). Fenanyl was temporarily discontinued if heart rate fell below 80 beats/min or the respiratory rate (RR) below 20 breaths/m. Fentanyl was reintroduced if agitation or tremor developed. Neonates received whole body TH using a cooling blanket (CureWrap™) connected to a cooling pump (Criticool, Belmont Medical Technologies, Somerville, Massachusetts), to maintain the rectal temperature at 33.5°C for 72 h. Neonates were then rewarmed by increasing the temperature of the blanket by 0.5°C per hour until the core body temperature reached 36.5°C.

### 2.2 Respiratory flow and Pes measurements

Flow was detected using a pneumotachograph (4,500 series, not heated, flow range 0–35 LPM; Hans Rudolph, Shawnee, Kansas) combined with a differential pressure transducer (SensorTechnics 144LU01D-PCB, SensorTechnics, Inc., Mansfield, CA), inserted into the facemask port. Pes, as a surrogate for pleural pressure, was measured with a single 1.3 mm (approximately 4 Ch) diameter solid-state pressure transducer at the catheter tip (CTO-1, Gaeltec, Dunvegan, Scotland) (23). Correct catheter placement in the lower third of the esophagus was confirmed using the occlusion technique (24). Flow and Pes signals were sampled simultaneously using a portable oscilloscope (PicoScope 4,824, Pico Technology, UK), and acquired for off-line analysis (25). Flow and Pes tracings were combined to obtain data for respiratory mechanics (dynamic compliance, Cdyn, and inspiratory and expiratory airway resistances, Rinsp, Rexp, respectively) and respiratory effort (esophageal pressure-time product per minute, PTPes/min) indices using specific routines (MATLAB® software), as previously reported (23, 25).

### 2.3 Recording protocol

Daily recordings of flow and Pes were made on day 1, 2, and 3 of TH and on day 5 of life during normothermia (a total of four days). Day 1 measurements were taken near the end of the day to enable neonates to adjust to hypothermia and to nearly resolve initial metabolic acidosis. The neonates who had not been treated with TH were studied on the 2nd day of life.

Before insertion of the Pes catheter, the nasogastric tube was removed and local suctioning was performed. All neonates were kept in a supine position with a roll under their shoulders. Most neonates showed hypertonicity in their arms and legs, and hypotonicity in their trunk muscles. To minimize interference with normal breathing and thus artifacts, flow recordings lasted 80–120 s. Once the Pes catheter had been correctly placed, the Pes trace was monitored and, if stable, it was recorded for twenty seconds. The pneumotachograph coupled-mask was placed on the neonate's face for 40 s, provided that respiration was steady, the mask was removed for 20 s to allow the neonate to rest. If discrepancies between the pre- and post-mask Pes shape were observed on the display, the mask was repositioned for an additional 40 s. If artifacts persisted, up to three additional sessions were allowed, otherwise the neonate was excluded from the study. Specifically, flow and Pes recordings were repeated if (a) they showed volume signal drift, (b) the flow signal was disturbed by neonate movement, (c) respiration was unstable (rising or falling), or (d) desaturation occurred. All cooled neonates were receiving fentanyl during measurements. Uncooled neonates with HIE received 0.5 mcg/kg fentanyl prior to Pes catheter insertion.

In limited cases, we used silicone mold putty to improve adhesion of the mask. All patients were assessed at the bedside in the PICU using the same instruments.

### 2.4 Classification of breathing patterns in braked or unbraked based on inspiration-to-expiration ratio

**Figure 1:**
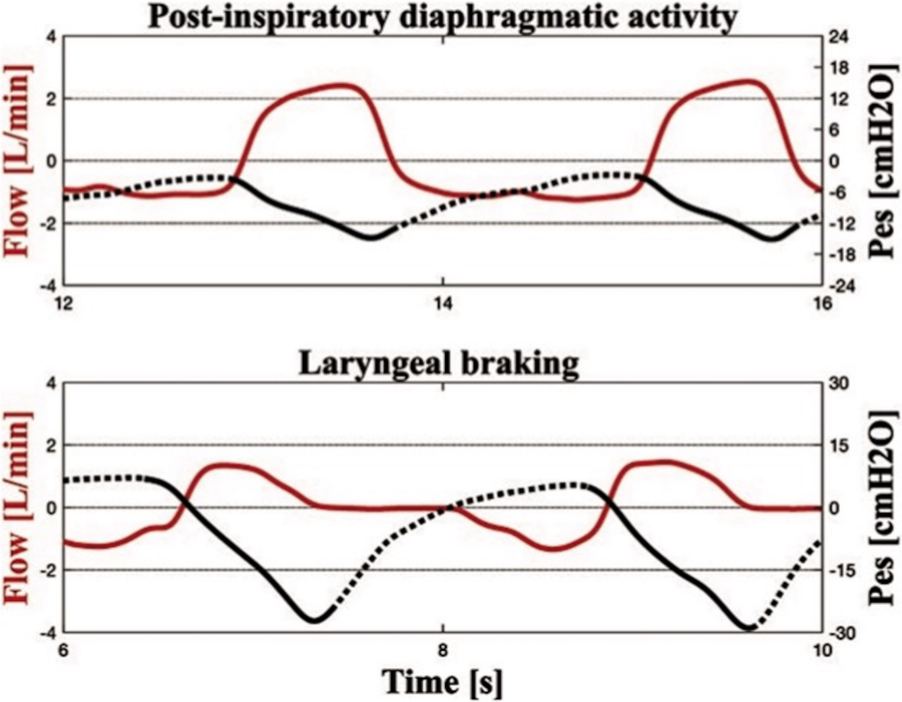
Simultaneous recordings of flow and esophageal pressure (Pes) during therapeutic hypothermia. In the upper panel, the Pes curve indicates the post-inspiratory diaphragmatic activity with a slowly increasing Pes curve during expiration. The expiratory resistance is 178.1 cm H_2_O/L/s. In the lower panel, the positive Pes curve at the end of expiration indicates laryngeal braking (expiratory resistance of 1,961.7 cm H_2_O/L/s).

As previously reported, neonatal breathing patterns are classified as braked or unbraked based on the inspiration-to-expiration (I:E) ratio. Unbraked breaths (I:E <1.5) have a shorter expiratory phase, while braked breaths (I:E ≥1.5) have a prolonged expiratory phase caused by either laryngeal adduction or post-inspiratory diaphragmatic activity. Braking may be considered due to diaphragmatic post-inspiratory activity if the Pes signal returns slowly to baseline after the descendent and Rexp is normal. On the other hand, braking may be considered laryngeal if the Rexp is high and the Pes signal returns rapidly to baseline after the descendent and becomes positive until expiration ends (Figure 1). Sighs were defined as tidal breaths that were approximately twice the mean Vt of the previous 5–10 breaths.

### 2.5 Statistical analysis

This is the first study of neonatal breathing patterns under TH for HIE. Therefore, no formal *a priori* sample size calculation could be performed. Nonetheless, the attained sample size aligned with that of other physiologic studies in the medical field. Our decision to include 20 neonates in the study was made based on similar physiologic research (14). To investigate the impact of therapeutic hypothermia (TH) on breathing patterns, we planned to enroll twenty neonates with perinatal asphyxia who were not treated with TH as controls. However, due to the slow recruitment rate of control neonates, we performed statistical analysis to compare breath types and the effect of TH vs. normothermia on the same breath type. Data are presented as numbers and percentages (%), median and interquartile range (IQR). Non-parametric methods (Mann-Whitney *U*-test) were employed to assess the differences in unbraked breaths between TH and normothermia. The Kruskal-Wallis test was utilized to investigate the variations among types of braked breaths during TH, and a *post hoc* analysis was conducted between pairs of types of breath. Correlation analyses between respiratory variables were performed using the Spearman's rank correlation test. A *p*-value of less than 0.05 was considered significant. Data analysis was performed using SPSS (v27.0; Lead Technologies, Chicago, IL).

## 3 Results

### 3.1 Characteristics of infants studied

Flow and Pes tracings were obtained from twenty neonates who received TH for HIE and from three neonates with perinatal asphyxia who did not meet all the criteria for TH. Three of the twenty cooled neonates were excluded from the final analysis because of poor signal quality on at least one of the four recording days (1–3, 5). The remaining seventeen had recordings suitable for further processing and analysis. All of the three uncooled neonates had good signals from the day of recording (day 2 of life). Demographic and clinical characteristics of the seventeen neonates who underwent TH for HIE are reported in Table 1. The clinical characteristics of the four infants who received TH for mild HIE are presented in Supplementary Appendix 1A and compared with the three infants who did not receive TH. Most infants had hypertonic arms and legs and hypotonic trunk muscles during TH. No signs of neurological decline were observed in any of the infants during the study period. Only one newborn (6%) who received TH had positive MRI findings of the brain parenchyma (millimeter-sized nodules in semi-oval white matter centers in T1 hypersignal). In four of the 17 infants who received TH, we observed only a small amount of subarachnoid or subdural hemorrhage.

**Table 1:** Clinical features of the 17 neonates under therapeutic hypothermia (TH) with hypoxic ischemic encephalopathy (HIE).

Characteristics number	TH neonates (17)
Gestational age, weeks median (IQR)	39.5 (0.7)
Birth weight, g median (IQR)	3,207.7 (183.8)
Male/Female	8/9
Fetal heart rate abnormalities, %	24
Stained amniotic fluid, %	35
Intrapartum adverse events, %	41
Cesarean delivery, %	41
Need for resuscitation at 10′, %	71
Intubation at birth, %	12
Apgar at 5′, median (IQR)	8.0 (6.25–8.75)
Base excess <1 h, mmol/L median (IQR)	−15.24 (2.9)
Sarnat score of 1, %	24
Mild HIE, %	24
EEG abnormalities on day 10, %	6
Brain abnormalities on MRI, %	6
AOAE test (refer), %	35
Retinal hemorrhage, %	35

EEG, electroencephalogram; AOAE, auditory otoacoustic emissions.

### 3.2 Breathing patterns definition based on expiratory flow shape

**Figure 2:**
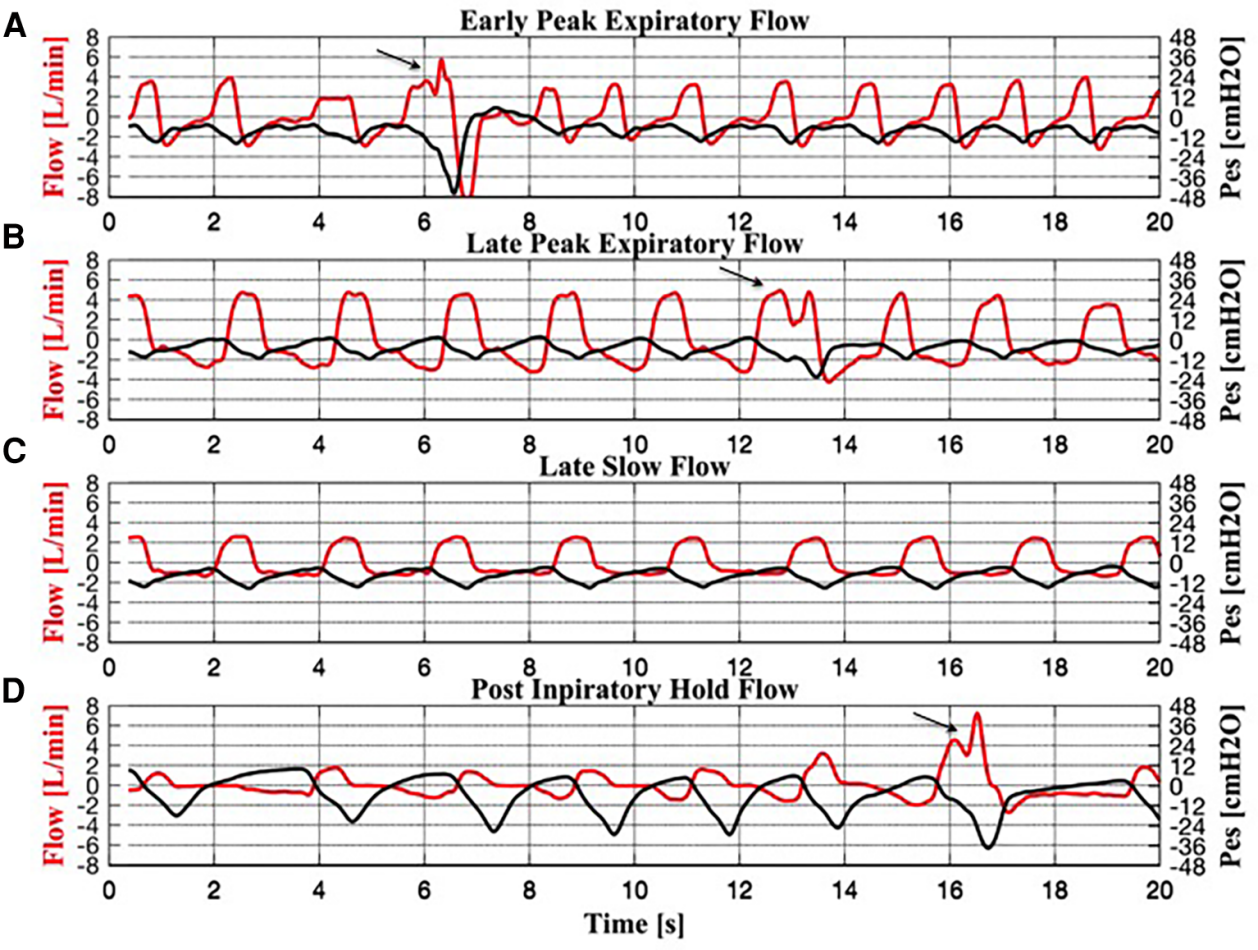
Simultaneous recordings of flow and esophageal pressure (Pes) for each braked breath. (**A**) Early peak expiratory flow pattern. (**B**) Late peak expiratory flow pattern. (**C**) Slow flow pattern. (**D**) Post-inspiratory hold flow pattern. Three sighs can be observed in panels (**A**), (**B**) and (**D**), indicated by an arrow.

**Figure 3:**
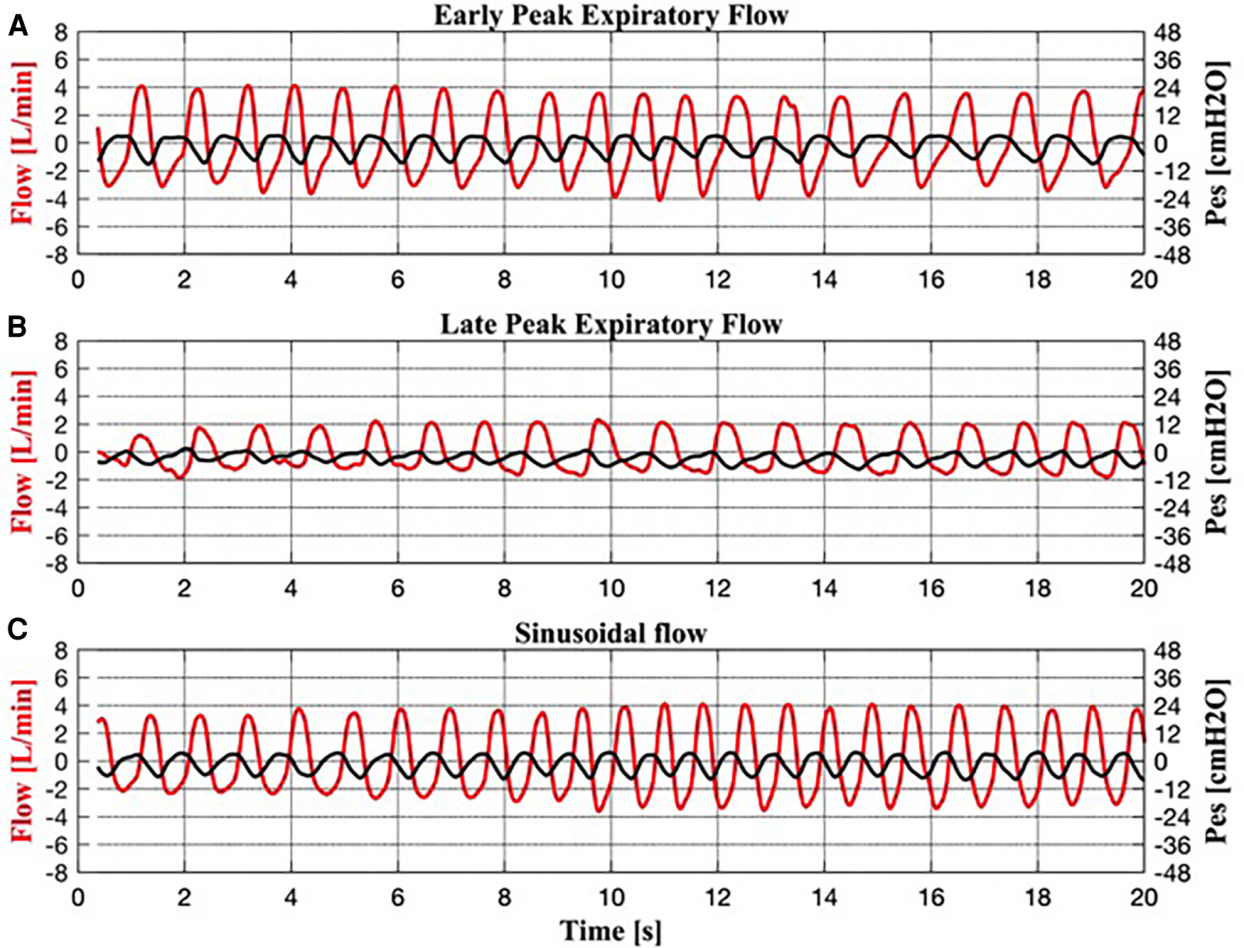
Simultaneous recordings of respiratory flow and esophageal pressure (Pes) for each unbraked breath. (**A**) Early peak expiratory flow pattern. (**B**) Late peak expiratory flow pattern. (**C**) Sinusoidal flow pattern.

The analysis of the expiratory flow tracings of the 17 infants under TH revealed four braked patterns (Figure 2) and three unbraked patterns (Figure 3). Each pattern showed low breath-to-breath variability in each 20-s recording window; the few discordant breaths were excluded, or analyzed separately (sighs). There was also enough consistency in the breathing patterns between the days of TH (Table 2).

**Table 2:** Breathing patterns of each infant cooled for hypoxic ischemic encephalopathy by day of life.

		Therapeutic hypothermia						Normothermia	
		Day 1		Day 2		Day 3		Day 5	
Patient N°	HIE severity	Expiratory braking	Flow pattern	Expiratory braking	Flow pattern	Expiratory braking	Flow pattern	Expiratory braking	Flow pattern
1	Mild	Yes	PiHF	Yes	PiHF	Yes	PiHF	No	Sinusoidal
2	Moderate	Yes	LPEF	Yes	LPEF	Yes	LPEF	No	Sinusoidal
3	Moderate	No	Obstructed	No	LPEF	No	LPEF	No	Sinusoidal
4	Moderate	No	LPEF	No	EPEF	Yes	LPEF	No	Sinusoidal
5	Mild	Yes	LPEF	Yes	LPEF	Yes	LPEF	No	Sinusoidal
6	Moderate	Yes	LPEF	Yes	EPEF	Yes	LPEF	No	LPEF
7	Moderate	Yes	EPEF	Yes	EPEF	Yes	EPEF	Yes	EPEF
8	Moderate	Yes	PiHF	Yes	PiHF	Yes	LPEF	Yes	EPEF
9	Moderate	Yes	LPEF	No	LPEF	Yes	LPEF	No	Sinusoidal
10	Mild	No	LPEF	Yes	EPEF	Yes	PiHF	No	Sinusoidal
11	Moderate	Yes	EPEF	Yes	EPEF	Yes	EPEF	Yes	EPEF
12	Moderate	Yes	EPEF	Yes	EPEF	Yes	EPEF	No	EPEF
13	Moderate	No	EPEF	Yes	PiHF	Yes	PiHF	No	EPEF
14	Moderate	No	Obstructed	No	Obstructed	No	Obstructed	No	LPEF
15	Mild	Yes	PiHF	Yes	PiHF	Yes	PiHF	Yes	EPEF
16	Moderate	Yes	EPEF	Yes	SF	Yes	SF	Yes	EPEF
17	Moderate	Yes	EPEF	Yes	EPEF	Yes	EPEF	No	Sinusoidal

N°, number; EPEF, early peak expiratory flow; HIE, hypoxic ischemic encephalopathy; LPEF, late peak expiratory flow; PiHF, post-inspiratory hold flow; SF, slow flow.

The braked flow patterns included:

Braked Pattern #1 (early peak expiratory flow, EPEF, Figure 2A) showed an expiratory flow that decreased rapidly to a peak, followed by a fairly exponential trajectory toward baseline. The corresponding Pes swing showed a slowly rising expiratory phase after a rapid descent.
Braked Pattern #2 (late peak expiratory flow, LPEF, Figure 2B) showed a slowly descending expiratory flow ending with a single flow peak late in expiration. The corresponding Pes swing showed a slowly rising expiratory phase.
Braked Pattern #3 (slow flow, SF, Figure 2C) showed an initial rapid expiratory flow soon followed by a steady slow flow that returned to baseline when expiration ended, without peaks. The corresponding Pes swing showed a slowly rising expiratory phase.
Braked Pattern #4 (post-inspiratory hold flow, PiHF, Figure 2D) showed a postinspiratory pause followed by a rounded peak shape expiratory flow. Expiration was immediately followed by an inspiration. In the PiHF breathing pattern, the ascending Pes signal crossed the baseline and became positive when expiration ended.

The unbraked flow patterns (I:E <1:1.5) included:

Unbraked Pattern #1 (Figure 3A) resembled the EPEF pattern in flow and Pes.
Unbraked Pattern #2 (Figure 3B) resembled the LPEF pattern in flow and Pes.
Unbraked Pattern #3 (Figure 3C) was characterized by sinusoidal inspiratory and expiratory flow and a high RR achieved by shortening the inspiratory and expiratory phases (I:E approximately 1:1). Similarly, the corresponding Pes showed a sinusoidal shape.

Each of the three uncooled infants exhibited all three patterns of unbraked breathing observed in cooled infants, including EPEF, LPEP, and sinusoidal flow patterns.

### 3.3 Respiratory characteristics of sighs

Recordings during TH showed several “sighs” (Figure 2). Sighs were characterized by high peak inspiratory flow (PIF) followed by single or multiple inspiratory peaks in the descending phase, and prolonged inspiratory time. Sighs had a long median (IQR) Ti [1.03 (0.89–1.13) s], a high median PIF [5.06 (3.86–6.09) L/min] and a large median Vt [49.90 (36.42–62.10) ml] and Vt/kg [15.64 (11.49–19.87) ml]. Sighs were associated with a large median Pes swing [23.48 (20.60–32.94) cmH_2_O] and a low median Rinsp [15.52 (10.37–27.99) cmH_2_O/L/s]. Median (IQR) PTPes/min was 257.03 [200.27–367.30) cmH_2_O*s/min]. Morphologically, sighs resembled the EPEF pattern. During normothermia, fewer sighs were observed compared to TH (0.9% vs. 7%).

### 3.4 Distribution of braked and unbraked patterns during TH and normothermia

**Figure 4:**
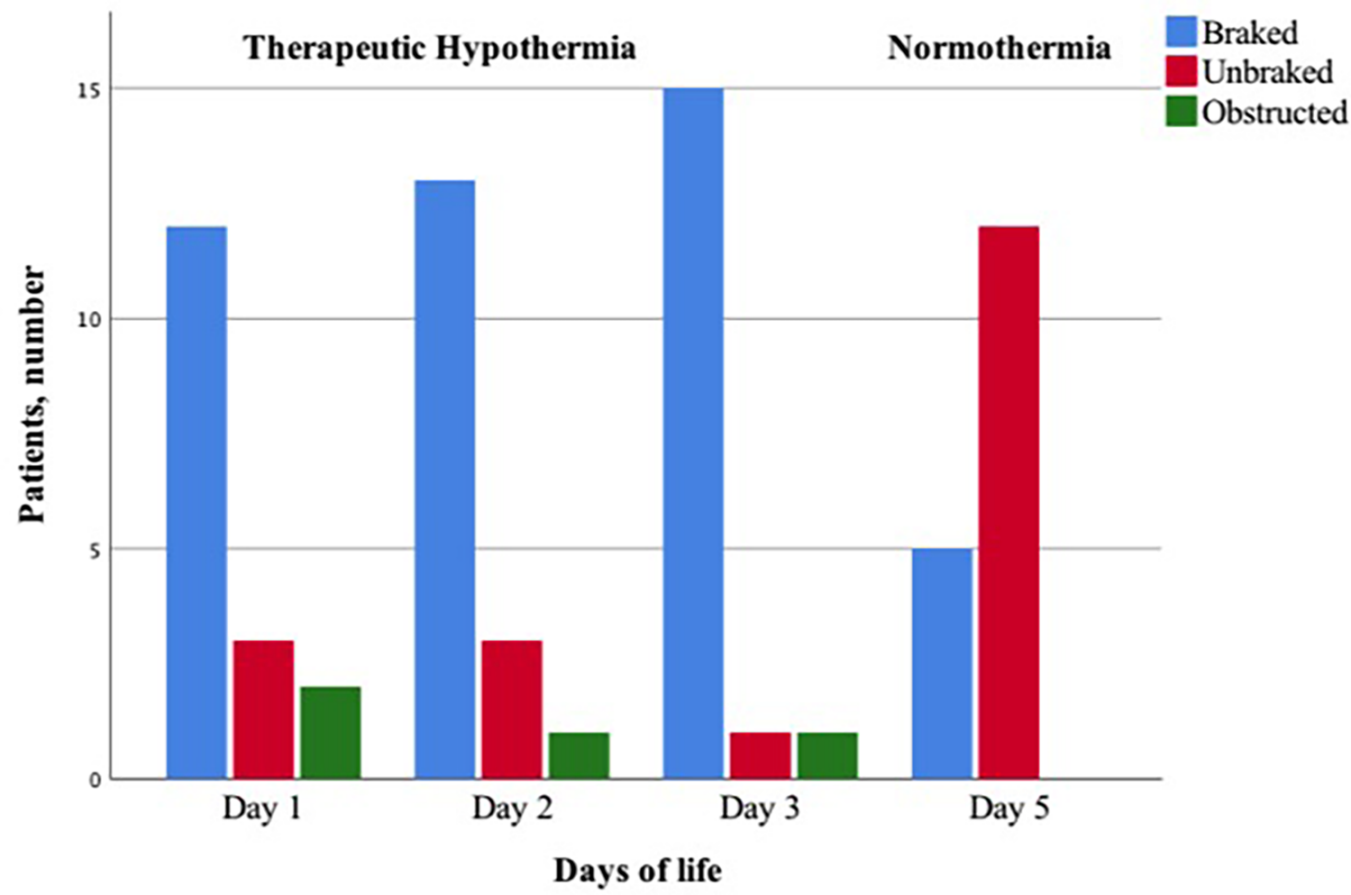
Proportion of infants with braked, unbraked, or obstructed respiratory flow pattern during therapeutic hypothermia and normothermia.

During TH day 1, 2, and 3, the percentage of infants with braked breaths increased, whereas at normothermia infants with unbraked breaths predominated, although five infants still presented the braked EPEF pattern (Table 2) (Figure 4). On day 1, 71% of infants showed a braked pattern with a slight prevalence of EPEF pattern (Table 2). On day 2, 76% of infants showed braked breaths with still a prevalence of EPEF, whereas on day 3, 88% showed the braked pattern (particularly the LPEF). Only two infants (11%) showed an obstructed pattern characterized by flow flattening on the inspiratory trace. Spontaneous pattern changes were rarely observed during each recording. The four infants with mild HIE showed the same patterns as those with moderate HIE, with a prevalence of PIHF (58%).

### 3.5 Comparisons of respiratory parameters between braked or unbraked patterns during TH and normothermia

The respiratory variables exhibited notable differences among the four braked flow patterns observed during TH (Table 3). The most braked type of breath was the slow flow, which demonstrated the longest Te, the lowest RR, and a considerable high Rexp. A similar pattern was observed in the PIHF type of breath, although the latter exhibited a lower ventilatory efficiency (lowest Vt/kg) than the other breathing patterns, despite a remarkable elevated cost of breathing (highest PTPes median values). The EPEF and LPEF exhibited comparable respiratory mechanics data and cost of breathing between each other. Nevertheless, the LPEF exhibited an exceedingly high respiratory efficiency, as indicated by the highest Vt/kg and Ve median values. According to the Pes shape and Rexp values, diaphragmatic post-inspiratory activity was the mechanism for expiratory deceleration in the EPEF, LPEF, and SF patterns. In contrast, vocal cord adduction was the mechanism in the PiHF pattern. The EPEF braked pattern was observed both under TH and normothermia after TH. Notably, there was a slight difference between the two, indicating more braking during TH. For example, RR was lower during TH [32.26 (26.91–41.02) breaths/min vs. 45.60 (36.77–55.10) breats/min, *p* < 0.001] than in normothermia and Vt was higher [24.67 (20.76–30.12) ml vs. 21.02 (19.62–25.39) ml, *p* = 0.03]. So was Vt/kg [7.80 (6.55–9.49) ml vs. 6.03 (5.63–7.29) ml, *p* = 0.001].

**Table 3:** Median (interquartile range) values of respiratory variables for each braked breathing pattern during TH; comparisons (Kruskal Wallis test with *post hoc* analysis) are made between breathing patterns.

Braked breaths during TH				
	#1 early peak expiratory flow	#2 Late peak expiratory flow	#3 Slow flow	#4 Post-inspiratory hold flow
N° of breaths	134	112	31	98
Ti:Te	1.77 (1.55–2.04)	1.93 (1.70–2.19)	2.08 (1.84–2.50)	2.02 (1.67–2.78)
Ti, s	0.67 (0.54–0.74)	0.74 (0.59–0.82)	0.76 (0.75–0.78)	0.56 (0.49–0.88)
Te, s	1.16 (0.97–1.42)	1.26 (1.15–1.68)	1.60 (1.40–1.89)* vs. #1	1.32 (1.05–1.59)
RR, bpm	32.26 (26.91–41.02)* vs. #3	29.97 (24.57–33.48)	25.43 (22.14–27.60)	28.04 (23.47–34.11)
Vt, ml	26.76 (20.77–30.12)* vs. #4	31.99 (25.15–36.85)* vs. #1	23.22 (21.35–24.21)	20.64 (18.48–27.09)
Vt/kg, ml	7.80 (6.55–9.50)* vs. #4	11.17 (8.77–12–86)* vs. #1,4	9.59 (8.81–10.01)* vs. #4	6.00 (5.38–7.88)
Ve, L/min	0.81 (0.64–0.97)* vs. #3,4	0.89 (0.76–1.02)* vs. #3,4	0.58 (0.51–0.65)	0.65 (0.48–0.74)
PIF, L/min	3.35 (2.91–4.10)* vs. #3	3.90 (3.01–4.83)* vs. #3	2.44 (2.20–2.61)	3.03 (2.42–4.02)
Cdyn, ml/cmH_2_O	3.07 (2.44–4.70)* vs. #4	3.69 (2.81–4.35)* vs. #4	3.03 (2.76–3.14)	2.26 (1.31–2.79)
Rinsp, cmH_2_O*s/L	36.57 (28.84–51.50)	32.22 (23.40–85.90)	55.18 (32.49–71.88)	157.03 (126.47–195.70)* vs. #1,2
Rexp, cmH_2_O*s/L	82.62 (60.39–103.34)	72.43 (45.54–138.01)	166.24 (108.64–250.15)* vs. #2	381.77 (245.37–1,345.50)* vs. #1,2
Pes swing, cmH_2_O	9.46 (7.79–10.95)	9.65 (7.94–14.82)	9.66 (8.79–11.75)	22.72 (19.67–27.37)* vs. #1,2,3
PTPes/min, cmH_2_O*s/min	116.35 (100.09–136.18)	120.85 (101.35–204.61)	123.05 (103.85–158.78)	300.73 (255.76–374.72)* vs. #1,2,3

Statistical significance is indicated by * (*p* < 0.05).

N°, number; Ti, inspiratory time; Te, expiratory time; RR, respiratory rate; PIF, peak inspiratory flow; Cdyn, dynamic compliance; Rinsp, inspiratory resistance; Rexp, expiratory resistance; Pes, esophageal pressure; Vt, tidal volume; Ve, minute volume; PTPes, esophageal pressure time product; TH, therapeutic hypothermia.

During therapeutic hypothermia (TH), unbraked breaths exhibited similar flow patterns to their counterparts during normothermia. These breaths included EPEF, LPEF, and sinusoidal flow. Overall, unbraked breaths during TH showed longer Te, lower RR, and lower Ve compared to normothermia. Vt/kg tended to be higher during TH and, with a few exceptions, Rinsp, Pesswings, and PTPes were lower suggesting reduced respiratory effort (Table 4).

**Table 4:** Median (interquartile range) values of respiratory variables for each unbraked breathing pattern recorded in the 17 infants during therapeutic hypothermia (TH) (days 1, 2, 3) and normothermia (day 5); comparisons (mann-whitney *U*-test) are made between the same types of breathing pattern.

	Unbraked breaths under TH			Unbraked breaths under normothermia		
	Early peak expiratory flow	Late peak expiratory flow	Sinusoidal flow	Early peak expiratory flow	Late peak expiratory flow	Sinusoidal flow
Breaths, N°	12	10	10	26	21	84
Ti:Te	1.44 (1.39–1.48)	1.39 (1.39–1.47)	1.24 (1.23–1.25)	1.32 (1.14–1.37)	1.23 (1.11–1.32)^††^	1.19 (1.09–1.31)
Ti, s	0.59 (0.53–0.74)	0.81 (0.78–0.82)	0.44 (0.43–4.44)	0.57 (0.52–0.61)	0.57 (0.55–0.61)^††^	0.42 (0.39–0.47)
Te, s	0.77 (0.74–1.09)	1.14 (1.11–1.16)	0.54 (0.54–0.55)	0.73 (0.64–0.79)	0.68 (0.66–0.75)^††^	0.50 (0.45–0.54)
RR, bpm	40.33 (39.96–41.66)	41.97 (40.92–42.42)	45.12 (45.04–45.19)	43.07 (42.01–46.49)	44.93 (43.14–47.29)^††^	45.08 (42.91–47.81)
Vt, ml	28.70 (20.37–32.09)	28.20 (22.72–28.61)	27.25 (25.91–28.59)	26.08 (22.26–26.78)*	27.71 (26.53–32.75)	22.61 (16.55–27.18)^‡^
Vt/kg, ml	9.04 (6.42–10.12)	9.84 (8.92–9.99)	8.18 (7.85–8.66)	6.04 (5.63–7.29)**	10.25 (9.86–12.01)^†^	6.68 (4.90–6.69)^‡^
Ve, L/min	1.04 (0.91–1.20)	0.89 (0.70–0.92)	1.63 (1.51–1.76)	1.20 (1.03–1.36)	1.33 (1.20–1.56)^††^	1.35 (1.09–1.54)^‡‡^
PIF, L/min	3.86 (3.66–4.94)	2.56 (2.44–2.88)	5.88 (4.91–6.85)	4.01 (3.09–4.31)**	4.16 (3.76–4.67)^††^	4.32 (3.67–5.37)^‡‡^
Cdyn, ml/cmH_2_O	3.67 (2.25–4.46)	3.03 (2.03–3.20)	2.77 (2.26–3.29)	3.63 (2.62–3.95)	4.25 (3.67–4.64)^††^	3.12 (2.06.3.63)
Rinsp, cmH_2_O*s/L	47.84 (32.10–56.04)	100.03 (81.01–110.07)	48.72 (41.30–56.13)	83.47 (42.28–91.66)*	86.70 (79.24–90.51)^†^	35.50 (27.46–49.85)^‡^
Rexp, cmH_2_O*s/L	70.09 (56.12–119.01)	44.51 (40.11–157.88)	99.76 (57.46–142.05)	79.38 (73.98–83.34)*	79.25 (72.36–82.32)	60.16 (48.19–77.60)^‡^
Pes Swing, cmH_2_O	9.21 (7.81–14.72)	7.27 (6.93–14.84)	15.24 (11.30–19.17)	10.59 (9.92–11.67)	11.18 (10.28–11.90)	9.78 (8.37–11.30)^‡‡^
PTPes/min, cmH_2_O*s/min	147.61 (114.64–225.28)	149.16 (136.89–228.11)	256.68 (201.16–312.19)	241.68 (171.88–251.52)	249.84 (224.64–275.93)^††^	172.76 (153.41–222.70)**

The symbols *, †, and ‡ indicate statistical significance for early peak expiratory flow, late peak expiratory flow, and sinusoidal flow, respectively. One symbol indicates *p* < 0.05, two symbols *p* < 0.01.

N°, number; Ti, inspiratory time; Te, expiratory time; RR, respiratory rate; PIF, peak inspiratory flow; Cdyn, dynamic compliance; Rinsp, inspiratory resistance; Rexp, expiratory resistance; Pes, esophageal pressure; PTPes, esophageal pressure time product; Vt, tidal volume; Ve, minute ventilation.

### 3.6 Correlations betweens respiratory variables in braked patterns

RR and Te showed the highest degree of correlation (−0.832, *p* < 0.0001). Significant correlations were also found between RR and Vt (−0.204, *p* = 0.037), RR and Vt/kg (−0.230, *p* = 0.019), RR and PIF (0.295, *p* = 0.002), whereas no statistically significant correlation was found between RR and PTPes/min. Vt and Vt/kg correlated with Ti (0.484, *p* < 0.001, 0.494, *p* < 0.001), with PIF (0.542, *p* < 0.001, 0.461, *p* < 0.001), and inversely with Rinsp (−0.278, *p* = 0.004, −0.350, *p* < 0.001). Pes swing correlated negatively with Vt and Vt/kg (−0.299, *p* = 0.002, −0.388, *p* < 0.001) and positively with Rinsp (0.725, *p* < 0.0001).

### 3.7 Breathing patterns in uncooled infants

In the three uncooled infants the majority of breaths were unbraked. Only 5% of breaths were breaked, none of which showed the SF or the PiHF pattern. Respiratory flow tracings occasionally showed sighs. The unbraked breaths were equally distributed among sinusoidal flow pattern, LPEF pattern, and EPEF pattern. Their respiratory characteristics are shown in Supplementary Appendix 2A.

## 4 Discussion

Our main finding is that infants under TH for HIE adapt their airflow behaviour to hypothermia induced-changes in respiratory timing, nearly mirroring the “expiratory breaking” patterns of healthy neonates shortly after birth. During TH, the frequency of expiratory braking increases over time, showing multiple flow patterns. All braked breaths, together with prolonged expiration, have low rates and large Vt, with few exceptions. Due to the favourable respiratory mechanics and low rates, braked breathing may provide an optimal ventilatory strategy during TH, which can increase EELV at low respiratory cost (26).

### 4.1 Respiratory patterns during TH

Previous studies have reported interrupted expiratory flow in healthy term and preterm infants up to 90 min after birth (18, 27). In newborns, braked breathing manifests as expiratory hold, slow expiration, grunting, and crying (14). Frequency peaks soon after birth and declines over the next few hours or days as the braked pattern is replaced by unbraked and rapid breathing, interspersed with a few braked breaths (18, 27, 28). Conversely, in infants under TH, the frequency of braked breaths increases over time, becoming the most prevalent pattern on TH day 3. Under physiological conditions, the upper airway control of expiratory resistance by means of vocal cord adduction is critical in the first hours after birth, favoring an increase in EELV and pulmonary fluid reabsorption (29). After the initial stage of the newborn's adaptation to the external environment, increased upper airway expiratory resistance is maintained only in neonates breathing at relatively low rates (18). When the EELV is above the resting volume, inspiratory effort increases, which is undesirable in neonates with high metabolic demands and high RR. Thus, it is not surprising that the braked breathing strategy is rapidly abandoned in healthy term infants, whereas it is adopted during TH when the metabolic demands and RR are low. Unlike normothermic neonates, in whom high RR contribute to an increase in EELV after the first days of life (30), in cooled infants TH related slow RR would give the lungs time to return to relaxation volume. Expiratory braking may compensate this mechanism by increasing EELV. This effect is relevant because breathing from a low lung volume would result in decreased oxygen stores, and may place infants at risk for possible airway closure leading to progressive atelectasis (17).

### 4.2 Factors supporting an expiratory braking pattern during TH

Despite studies on EELV in cooled infants are scanty, experimental evidence suggests that this population may be prone to low EELV (31). Asphyxia and hypothermia promote alterations in alveolar epithelial tight junctions leading to intrapulmonary fluid accumulation (31). Thus, cooled neonates may require additional time to clear pulmonary fluid beyond the first few hours of life. In this pathological context, infants with HIE may experience challenges in ventilating their lungs and may require longer “hold maneuvers” than healthy infants. Studies in healthy term newborn infants have shown that crying is the most important hold maneuver (14). Birth asphyxia and mild sedation with fentanyl during TH might reduce the typical crying in the first hours of life, which generates high intrathoracic pressures, helps clear fluid from the lungs, and facilitates ventilation. In this context, a series of braked breaths with high Vt and occasional sighs may compensate for the absence of crying. Nicholas et al. have shown experimentally that a single deep breath releases surfactant into the alveolar compartment and re-expands atelectatic alveoli (32). Our findings show that most braked breaths had a large Vt, which is consistent with previous studies on mechanically ventilated, cooled infants for HIE. In these studies, Vt was monitored to remain within a predefined range, but there was an increase in Vt when the target temperature was achieved and a decrease during rewarming (33).

Besides low RR and intrapulmonary retention of lung fluid, breaked breaths could also increase owing to changes in blood gases during TH. Previous studies have shown that hypocapnic hypoxia increases the discharge frequency in the phrenic nerve fibers and prolongs post-inspiratory diaphragmatic activity (4). During prolonged expiration and post-inspiratory hold, the relative distribution of inspired gas can be improved to enhance carbon dioxide elimination in case of reduced RR (34).

### 4.3 Respiratory mechanics during TH

Our study presents new insights into lung mechanics during TH. Experimental and clinical reports suggest that ventilatory mechanics depends closely on changes in body temperature (35, 36). Although deep hypothermia resulted in decreased pulmonary compliance in anesthetized ventilated lambs (35), mild hypothermia had no similar effects in ventilated adult patients after cardiac arrest (37). In a clinical study of respiratory function variables in ventilated neonates undergoing whole-body hypothermia, Dassios and Austin showed that static compliance might increase during whole-body hypothermia in infants with HIE, improving oxygenation and ventilation (33). Curran and Halloran have demonstrated that moderate upper airway cooling reduces resistance in young guinea-pigs owing to increased geniohyoid muscle activity (i.e., vocal cord abduction). The authors also suggest that the fall in upper airway resistance is a direct effect of cooling on upper airway mucosal blood flow (38). Taken together, these results support our findings of low Rinsp in most braked breaths during TH. Our study on spontaneously breathing infants cooled for HIE shows an increased Vt without causing hyperventilation. This explains how cooled infants maintain adequate ventilation despite low RR and minute ventilation. Notably, despite the increase in RR upon rewarming to normothermia, Vt remained unexpectedly high in certain types of breaths. During the rewarming process, there is a significant increase in carbon dioxide blood levels due to increased metabolic demands, oxygen consumption, and carbon dioxide production (39). Previous experiments conducted by Torbati et al. on anesthetized spontaneously breathing rats, the authors observed a Vt of 7.6 ± 3.1 ml/kg before hypothermia and 10.3 ± 4.2 ml/kg after rewarming (39). In our study, carbon dioxide levels returned to normal on day 5 compared to the last day of TH, when they were slightly elevated. We speculate that increasing both Vt and RR may be useful to eliminate excess carbon dioxide in accordance with increasing metabolic demands.

### 4.4 Breathing effort during expiratory braking

As suggested by the low PTPes/min values, expiratory braking results in a cost-effective breathing strategy for cooled infants. The decrease in Rinsp may be an explanation for the low PTPes/min. It is worth noting that the PiHF pattern is an exception to this trend. Theoretically, if the braking mechanism involves vocal cord adduction, it should help avoid muscle fatigue while allowing for adequate gas exchange (6, 26), whereas, if the braking mechanism involves the recruitment of accessory respiratory muscles or the crural diaphragm, as occurs during post-inspiratory diaphragmatic activity, increased respiratory effort would be expected. The behavior of vocal cords (partially adducted during inspiration and fully adducted during expiration) stimulated either by brain injury or laryngeal cooling (40) causes a reduction in the dilating effect of the posterior cricoarytenoid muscle on the vocal cords during inspiration (40). As a result, Rinsp becomes high, as does Pes, whereas Vt becomes low. This can be seen most clearly in the PiHF pattern. During expiration, the nearly complete closure of the vocal cords results in air being trapped in the lungs and the need to increase intrathoracic pressure (i.e., Pes) to expire.

### 4.5 Clinical implications

Our findings, taken together, offer guidance for clinical decisions regarding the need for respiratory support in infants with healthy lungs undergoing TH. For neonatologists who do not routinely intubate infants on TH, our data on the nature of spontaneous breaths suggests that clinical or subclinical breathing issues may occur as TH progresses in time because of an increase of low-rate breaths. In this scenario, the patient may benefit from ventilatory support, such as continuous positive airway pressure or high-flow nasal cannula, to support expiration and avoid unnecessary oxygen consumption. The breaths we need to carefully identify are the PiHF and the SF breathing. Even without pneumotachography, this type of breathing is indicated by a pause between inhalation and exhalation and, in the most severe cases, increased chest retractions.

For individuals who require intubation and mechanical ventilation for safety reasons, it is important to note that mechanical ventilation with deep sedation may increase the risk of hypocapnia in infants compared to spontaneous breathing. Increased sedation can significantly reduce tremors and shivering, leading to a decrease in metabolic rate and carbon dioxide production, ultimately reducing the need for ventilation. To prevent ventilation-induced hypocapnia, it is necessary to set low-minute volume ventilation.

### 4.6 Study limitations

Our study has limitations. We studied a small number of uncooled infants with less severe HIE than those who received TH. Also, due to the small number of controls and infants with mild HIE, and statistical analysis could not distinguish whether the observed changes in breathing patterns were due to the severity of asphyxia, hypothermia, or even fentanyl administration. Unfortunately, this area of research does not have a large number of ideal control infants for study. We therefore considered to slightly modify the study design by limiting comparisons to the first aim of the stidy that was describing and classifiyning the type of breaths and study their characteristics and differences between one another.

We studied infants only for short periods. We might have observed more differences in breathing patterns if we had used longer recordings. Tidal breathing measurements in neonates are limited to short-term assessments due to the added dead space and resistance of the face-mask and the pneumotachograph. Although the flow-through technique has been introduced to reduce dead space (41), and potentially increase measurement duration, even the small resistance of the pneumotachograph may be problematic leading to variations of RR or Vt.

### 4.7 Conclusion

This study is the first report to characterize the spontaneous breathing pattern of infants with HIE during TH and normothermia after rewarming. Our results provide evidence that the breathing pattern during TH is predominantly braked, whereas the unbraked pattern is generally restored after rewarming. In the context of low rates, it is likely that slowed expiration is used to preserve lung volume. Considering the low respiratory effort, it is appropriate to refrain from assisting spontaneous breathing. However, if the PiHF pattern becomes predominant, it may be necessary to provide ventilatory support.

## Data Availability

The raw data supporting the conclusions of this article will be made available by the authors, without undue reservation.
