# Supplementary material for: Expiratory braking defines the breathing patterns of asphyxiated neonates during therapeutic hypothermia
Source: Front Pediatr. 2024 May 20;12:1383689. doi: 10.3389/fped.2024.1383689 (PMC11146197; doi:10.3389/fped.2024.1383689)
Supplement: Supplementary file 1 [file Table1.docx]

**APPENDIX 1**

**Table 1A.** Breathing patterns of infants with mild HIE who received TH and infants with perinatal asphyxia (Sarnat score of 1) (days 1, 2, 3) who did not receive TH (Controls) (day2).

| **Type of breath** | **Mild HIE during TH** | **Controls** |
| --- | --- | --- |
| **N° of infants** | **(4)** | **(3)** |
| Expiratory braking | 11/12 | 0 |
|  |  |  |
| EPEF, braked | 0 | 0 |
| EPEF, unbraked | 1/12 | 1/3 |
| LPEF, braked | 4/12 | 0 |
| LPEF, unbraked | 0 | 1/3 |
| SF | 0 | 0 |
| PiHF | 7/12 | 0 |
| Sinusoidal flow | 0 | 1/3 |

N°, number; EPEF, early peak expiratory flow; HIE, hypoxic ischemic encephalopathy; LPEF,

late peak expiratory flow; PiHF, post-inspiratory hold flow; SF, slow flow

**Table 2A.** Median (interquartile range) values of respiratory variables

for each unbraked breathing pattern recorded in the 3 uncooled infants

(day 2 of life). * p < 0.05, ** p < 0.01 (LPEF vs Sinusoidal Flow)

| **RESPIRATORY CHARACTERISTICS OF UNBRAKED BREATHS IN CONTROL INFANTS** | | | |
| --- | --- | --- | --- |
| **Respiratory**  **Variables** | **Type of breath** | | |
|  | **EPEF** | **LPEF** | **SinF** |
| Breaths, N° | 36 | 44 | 62 |
| Ti:Te | 0.92  (0.88-0.97) | 0.98  (0.88-1.15) | 0.90  (0.84-0.92) * |
| Ti, s | 0.53  (0.50-0.57) | 0.51  (0.49-0.56) | 0.54  (0.50-0.58) |
| Te, s | 0.50  (0.48-0.51) | 0.50  (0.47-0.56) | 0.49  (0.47-0.50) |
| RR, bpm | 52.01 (50.72.53.20) | 50.45  (46.58-53.23) | 52.51  (51.96-54.30) * |
| Vt, ml | 14.57  (13.26-15.53) | 13.95  (12.91-15.64) | 14.18  (13.48-15.15) |
| Vt/kg, ml | 4.46  (4.05-4.46) | 4.19  (3.8-4.65) | 4.34  (4.13-4.63) |
| Ve, L/min | 0.83  (0.80-0.90) | 0.80  (0.74-0.94) | 0.81  (0.79-0.89) |
| PIF, L/min | 2.23  (2.16-2.37) | 2.25  (2.13-2.47) | 2.21  (2.07-2.34) |
| Cdyn, ml/cmH_2_O | 2.76  (2.63-2.90) | 2.75  (2.58-2.84) | 2.75  (2.66-2.88) |
| Rinsp, cmH_2_O*s/L | 79.93  (73.54-85.85) | 69.21.  (56.20-82.06) | 81.22  (72.64-100.89) * |
| Rexp, cmH_2_O*s/L | 46.63  (39.87-51.71) | 42.65  (36.03-51.51) | 40.59  (38.36-51.59) |
| Pes Swing, cmH_2_O | 7.79  (7.42-8.01) | 7.44  (6.94.7.89) | 7.79  (7.42-7.95) |
| PTPes/min, cmH_2_O*s/min | 171.71  (162.04-181.40) | 159.16  (133.03-178.54) | 178.54  (165.81-182.44) * |

N°, number; Ti: inspiratory time, Te: expiratory time; RR: respiratory rate, PIF: peak inspiratory flow;

Cdyn: dynamic compliance; Rinsp: inspiratory resistance; Rexp: expiratory resistance; Pes: esophageal pressure;

PTPes: esophageal pressure time product; Ve, minute ventilation; Vt, tidal volume;

EPEF: early peak expiratory flow; LPEF: late peak expiratory flow; SinF, sinusoidal flow.
